# The epidemiology of dependency among urban-dwelling older people in the Dominican Republic; a cross-sectional survey

**DOI:** 10.1186/1471-2458-8-285

**Published:** 2008-08-13

**Authors:** Daisy Acosta, Ruth Rottbeck, Guillermina Rodríguez, Cleusa P Ferri, Martin J Prince

**Affiliations:** 1 Internal Medicine Department, Geriatric Section, Universidad Nacional Pedro Henriquez Ureña (UNPHU), Santo Domingo, Dominican Republic; 2 Charité Universitätsmedizin Berlin, Freie Universität and Humboldt Universität Berlin, Germany; 3 Dirección General de Salud Pública. Ministerio de Protección Social (6th District), Santo Domingo, Dominican Republic; 4 Section of Epidemiology, Health Service and Population Research Department, Institute of Psychiatry, King's College London, London, UK

## Abstract

**Background:**

Demographic ageing, and the health transition will soon lead to large increases in the number of dependent older people in low and middle income countries. Despite its importance, this topic has not previously been studied.

**Methods:**

A cross sectional catchment area one-phase survey of health conditions, dependency, care arrangements and caregiver strain among 2011 people aged 65 years and over in Santo Domingo, Dominican Republic

**Results:**

7.1% of participants required much care and a further 4.7% required at least some care. The prevalence of dependency increased sharply with increasing age. Dependent older people were less likely than others to have a pension and much less likely to have paid work, but no more likely to benefit from financial support from their family. Needing much care was strongly associated with comorbidity between cognitive, psychological and physical health problems. However, dementia made the strongest independent contribution. Among those needing care, those with dementia stood out as being more disabled, as needing more care (particularly support with core activities of daily living), and as being more likely to have paid caregivers. Dementia caregivers experienced more strain than caregivers of those with other health conditions, an effect mediated by behavioural and psychological symptoms.

**Conclusion:**

Dependency among older people is nearly as prevalent in Dominican Republic as in developed western settings. Non-communicable diseases, particularly dementia are the main contributing factors. Attention needs to be directed towards the development of age-appropriate healthcare, a long-term care policy, and mechanisms for ensuring the social protection of older persons.

## Background

In the world's developing regions people are living longer, and having fewer children. High fat diets, smoking and sedentary lifestyles are becoming more common. Chronic non-communicable diseases (NCDs) linked to ageing – heart disease, stroke, cancer and dementia – are much more in evidence, and beginning to be recognized as a public health priority [1]. While cancer and heart disease contribute mainly to mortality, much of the burden of other NCDs (dementia, mental disorders, diabetes and stroke) arises instead from years lived with disability)[2]. Chronic disability, and the conditions that contribute to it have received comparatively little attention, in research, policy or practice.

Disability, as defined by the World Health Organization (WHO), refers to difficulties in carrying out an activity due to increased effort, discomfort or pain, slowness or changes in the way the activity is performed. Dependency, defined as 'the requirement for frequent help from other people, beyond what would be expected by virtue of family or social ties' [3] stems from disability, but disability may occur without dependency.

Estimates from the WHO Global Burden of Disease project (where both disability levels and needs for care were inferred from diagnoses) suggest that the total population prevalence of dependency is similar worldwide, varying only from 4.4% to 5.1% by region, and will increase only marginally by 2050 [4]. However, this overall stability masks a substantial shift in the profile of dependency, occurring mainly in low and middle income countries (LAMIC), and linked both to rapid demographic ageing and the health transition. The proportions of dependent persons who are aged 60 and over will increase between 2000 and 2050, from 21% to 30% in sub-Saharan Africa, from 23% to 44% in India, from 23% to 47% in Latin America, from 30% to 60% in China, and from 45% to 61% in developed countries [5]. Over this period numbers of dependent older people are forecast to quadruple in most LAMIC, while numbers of dependent younger people remain relatively stable. Therefore, in all world regions dependency is rapidly becoming a problem associated with ageing processes, particularly non-communicable disease morbidity.

Dependency is an important outcome for policy and practice. Dependent older people need care and support, which, typically, means a family member, friend or neighbour. This support may or may not be forthcoming. Caregivers can be tied to their role, with little or no respite. In developed countries caregiving is consistently associated with role strain and a high prevalence of anxiety and depression [6]. There is also accumulating evidence of adverse effects on caregiver health [7] including increased mortality [8]. 10/66 Dementia Research Group pilot studies in 24 LAMIC centres indicated that in the absence of formal support services dementia caregivers often have to cut back on paid work to care or face the costs of hiring paid help [9]. There are no compensatory benefits [9]. Since dependent older people often live in multigenerational households with their caregiver and their caregiver's children, caregiver psychological and financial strain could have wider negative impacts on, for example, child health and development, retention in education, and poverty prevention.

The 10/66 Dementia Research Group has conducted comprehensive population-based cross-sectional surveys of catchment areas in ten developing countries (India, China, Nigeria, Cuba, Brazil, Venezuela, Mexico, Peru, Argentina and the Dominican Republic) [10]. This new resource will allow us to estimate the prevalence of dependency, and the relative contribution of different NCDs to needs for care in the general older population. We are also now able to characterise care arrangements for people with dementia in representative population-based samples, comparing and contrasting these with those needing care in the context of other health conditions. This report focuses on a survey of five catchment area districts in Santo Domingo, the capital of the Dominican Republic.

## Methods

The Dominican Republic shares the Caribbean island of Hispaniola with Haiti. The population is 9.4 million, and 0.5 million (5.7%) are aged 65 and over [11]. Life expectancy is 71 years for men and 75 for women. The Dominican Republic is one of the poorest of the 10/66 Latin American countries, with a per capita GDP (PPP) of US$ 9,200. In common with other countries in the region there are high levels of income inequality (a Gini index of 51.6). Forty two percent of the population live below the poverty line, one third of these in extreme poverty. Pension coverage, at only 18% of the economically active population, is one of the lowest in Latin America. Extensive reforms have been proposed, and are in the process of implementation. Community health care is provided by the government through the system of 'primary attention units'. Consultations are free, but medicines must be paid for. Despite low medical insurance coverage, private healthcare is widely used instead. The Dominican Republic has only twenty psychiatrists, twenty psychologists, and two neurologists per million population.

Study design and catchment area: A one-phase cross-sectional whole population catchment area survey in geographically defined districts in Santo Domingo. The survey protocol is described in detail in an open access publication [10]. Precision calculations indicated that an overall sample of 2,000 would allow estimation of a typical dementia prevalence of 2.5% with a precision of ± 0.9%. Atypical middle-class or high-income areas were avoided. The catchment areas selected were Villa Francisca, San Carlos, San Antón, Mejoramiento Social and Santa Barbara. After defining the boundaries, mapping was carried out to identify and locate households. Possible participants (inclusion criterion age 65 and over) were identified. Age was formally determined on revisit for the interview by comparing stated age according to participant and informant, and official records if available. Irreconcilable discrepancies of three or more years were settled using an event calendar approach. Participants were recruited following informed consent or on the basis of a relative's agreement in case of lack of capacity for consent due to dementia. Interviews were carried out in participants' own homes. All participants received the full assessment lasting approximately two to three hours. Ethical approval for the survey was provided by the research ethics committee for the Institute of Psychiatry, King's College London, and the Bioethics National Committee for Research in the Dominican Republic.

### Measures

#### 1) Outcome – dependency

The interviewer used a series of open ended questions in their interview with a key informant, to establish the presence or absence of dependency:

a) Who shares the home with the participant?

b) What kind of help does the participant need – inside of the home? – outside of the home?

c) Who, in the family, is available to care for the participant?

d) What help do you provide?

e) Do you help to organise care and support for the participant?

f) Is there anyone else in the family who is more involved in helping than you? What do they do? What about friends and neighbours? What do they do?

On the basis of the answers to these questions, the interviewer defined the family network, established if the older person needed and got any care from family members or others, identified who was responsible for organising and providing 'hands on' care, and if the informant was one of the main caregivers. Finally, they coded whether the older person required a) no care, b) care, some of the time or c) care, much of the time.

#### 2) Health conditions contributing to dependency

a) Dementia according to either the 10/66 dementia diagnosis algorithm [12] or DSM IV dementia criterion [13].

b) Physical illness. Self-reported stroke, and having three or more of 11 limiting physical impairments (arthritis or rheumatism; eyesight problems; hearing difficulty or deafness; persistent cough; breathlessness, difficulty breathing or asthma; high blood pressure; heart trouble or angina; stomach or intestine problems; faints or blackouts; paralysis, weakness or loss of one leg or arm; skin disorders such as pressure sores, leg ulcers or severe burns) [14]

c) Depression – ICD 10 depressive episode (mild, moderate or severe), derived using a computerised algorithm applied to a structured clinical interview, the Geriatric Mental State [15].

#### 3) Characterisation of those with dependency

a) Disability Activity limitation and participation restriction measured by the WHO-DAS II [16], specifically developed by the WHO as a culture-fair assessment tool for use in cross-cultural comparative epidemiological and health services research.

b) Care arrangements (only assessed among those needing care)

Time spent by the caregiver in the last 24 hours in specific caregiving activities [17]; communicating, using transport, dressing, eating, looking after one's appearance, and supervising,

c) Impact of providing care on caregivers (only assessed among those needing care)

Caregiver perceived strain – the Zarit Burden Interview [18-20] with 22 items that assess the caregiver's appraisal of the impact their involvement has had on their lives.

Economic strain – the extent to which the caregiver had cut back on or stopped work in order to provide care, and paid care inputs

d) Caregiver mental health – the Self Reporting Questionnaire 20 [21,22].

e) Behavioural and Psychological symptoms of dementia; the Neuropsychiatric Inventory, NPI-Q [23].

The assessments of care arrangements and caregiver strain were applied and refined in the previous 10/66 Dementia Research Group pilot studies, conducted in 26 LAMIC centres, including the Dominican Republic. The Zarit Burden Interview was found to have robust cross-cultural measurement properties [9,24], but has not been specifically validated for this population. The NPI-Q has been previously validated in Spanish [25], but we are not aware of any previous use in the Dominican Republic.

### Analyses

1) We report the prevalence of dependency (needing some care, needing much care, needing any care) by age and gender using Stata 9.2 survey commands to generate robust standard errors and 95% confidence intervals, taking account of household clustering.

2) We describe the sociodemographic characteristics, economic circumstances and health status of the sample by levels of dependency. We used a Poisson regression model (adjusted for household clustering) to estimate the independent associations of dementia, three or more physical impairments, stroke and ICD-10 depression with needing much care, controlling for age, gender, education and marital status. The resulting prevalence ratios, together with the prevalence of the exposure were used (STATA aflogit command) to calculate population attributable prevalence fractions (PAPFs) as an index of the salience of each health condition to the prevalence of dependency at the population level.

3) Among those needing care, we compare the health characteristics, care inputs, and indicators of caregiver strain between those with dementia (and their caregivers) and those with other health conditions (and their caregivers).

4) We generated a general linear model to estimate the independent contributions of dementia, depression, physical impairments and stroke to caregiver strain measured using the Zarit Burden Interview, controlling for the main sociodemographic characteristics of the care recipient and the caregiver, and their relationship. The proportion of the variance accounted for by these factors was estimated before and after adding, separately, time spent assisting with activities of daily living, and the severity of behavioural and psychological symptoms in the care recipient as potential mediating variables in the association between dementia and caregiver strain.

## Results

### Sample characteristics

Door-knocking of the five catchment areas yielded 2117 persons eligible for the study; 2011 (95%) provided informed consent and were interviewed. The principal characteristics of the participants are provided in Table 1. Their median age was 74.0 years (interquartile range 69.0 to 80 years, total range 65 to 104 years). Two thirds of the participants were female (65.8%). The large majority (70.3%) had not completed primary education. Living alone was unusual (12.6%); most lived in two to four person households. Many were separated or divorced (23.1%), with only 29.1% currently married.

**Table 1:** General characteristics of the sample, by dependency status

		Total sample N = 2011 N (%)	Dependent (needs care some or much of the time) N = 237 N (%)	Not dependent (does not need care) N = 1864 N (%)	Statistical test	p-value
Age	65–69 years	533 (26.5%)	17 (7.2%)	514 (29.0%)	χ^2 ^= 125.8 (1 df)	<0.001
	70–74 years	520 (25.9%)	41 (17.3%)	478 (27.0%)		
	75–79 years	397 (19.7%)	37 (15.6%)	360 (20.3%)		
	80 + years	561 (27.9%)	142 (59.9%)	418 (23.6%)		
Gender	Female	1324 (65.8%)	173 (70.0%)	1149 (65.0%)	χ^2 ^= 6.0 (1 df)	0.02
Education	None	392 (19.5%)	61 (26.9%)	331 (18.8%)	χ^2 ^= 4.5 (1 df)	0.03
	Some	1022 (50.8%)	113 (49.8%)	907 (51.5%)		
	completed primary	370 (18.4%)	32 (14.1%)	336 (19.1%)		
	Completed secondary	135 (6.7%)	10 (4.4%)	125 (7.1%)		
	completed tertiary	73 (3.6%)	11 (4.8%)	62 (3.5%)		
Marital status	Never married	139 (6.9%)	14 (6.0%)	125 (7.1%)	χ^2 ^= 16.6 (3 df)	0.001
	Married	586 (29.1%)	49 (21.0%)	534 (30.4%)		
	Widowed	806 (40.1%)	122 (52.4%)	683 (38.8%)		
	Divorced	465 (23.1%)	48 (20.6%	417 (23.7%)		
Lives alone		254 (12.7%)	13 (5.5%)	241 (13.6%)	χ^2 ^= 12.5 (1 df)	<0.001
Household size	Median (interquartile range)	2 (1–4)	3 (1–5)	2 (1–4)	Z = -3.1	0.002
Sources of income (not mutually exclusive)	Government or occupational pension	610 (30.4%)	57 (24.1%)	553 (31.2%)	χ^2 ^= 5.1 (1 df)	0.02
	Family transfers	582 (29.0%)	65 (27.4%)	517 (29.2%)	χ^2 ^= 0.3 (1 df)	0.57
	Disability pension	8 (0.4%)	6 (2.5%)	2 (0.1%)	χ^2 ^= 30.8 (1 df)	<0.001
	Paid work	165 (8.2%)	1 (0.4%)	163 (9.4%)	χ^2 ^= 21.7 (1 df)	<0.001
Household assets	1^st ^quarter – least	648 (32.2%)	87 (36.7%)	559 (31.6%)	χ^2 ^= 12.5 (1 df)	0.097
	2^nd ^quarter	444 (22.1%)	51 (21.5%)	392 (22.1%)		
	3^rd ^quarter	733 (36.4%)	81 (34.2%)	651 (36.8%)		
	4^th ^quarter – most	186 (9.2%)	18 (7.6%)	168 (9.5%)		

### Prevalence of dependency

Needs for care were identified in 237 participants (11.8%), of whom 94 (4.7%) were rated as needing some care and 143 (7.1%) much care. Prevalence of all levels of dependency increased with age (see Table 2), linearly for men, and exponentially among women. The prevalence of dependency was higher among men at younger ages, and among women in those over 80 years old.

**Table 2:** Prevalence (%) of needs for care with 95% confidence intervals, by age and gender

	65–69	70–74	75–79	80+	Total
No care					

Women	97.4 [95.0,98.6] n = 334	91.6 [88.1,94.2] 91.7 n = 296	92.1 [88.2,94.8] n = 244	70.4 [65.7,74.7] n = 276	86.9 [85.0,88.6] n = 1150
Men	95.7 [91.7,97.9] n = 179	92.8 [88.2,95.7] n = 181	87.9 [81.1,92.4] n = 116	84.5 [78.2,89.3] n = 142	90.6 [88.2,92.6] n = 618
Total	96.8 [94.9,98.0] n = 514	92.1 [89.5,94.1] n = 478	90.7 [87.4,93.2] n = 360	74.6 [70.9,78.1] n = 418	88.2 [86.7,89.5] n = 1770

Some care					

Women	0.9 [0.3,2.7] n = 3	4.0 [2.3,6.8] n = 13	3.8 [2.0,6.9] n = 10	10.5 [7.7,14.0] n = 41	5.1 [4.0,6.4] n = 67
Men	2.1 [0.8,5.6] n = 4	2.6 [1.1,6.0] n = 5	3.8 [1.6,8.8] n = 5	7.7 [4.5,12.9] n = 13	4.0 [2.7,5.7] n = 27
Total	1.3 [0.6,2.7] n = 7	3.5 [2.2,5.4] n = 18	3.8 [2.3,6.2] n = 15	9.6 [7.4,12.4] n = 54	4.7 [3.8,5.7] n = 94

Much care					

Women	1.7 [0.8,3.8] n = 6	4.3 [2.6,7.2] n = 14	4.2 [2.3,7.3] n = 11	19.1 [15.6,23.3] n = 75	8.0 [6.7,9.6] n = 106
Men	2.1 [0.8,5.6] n = 4	4.6 [2.4,8.6] n = 9	8.3 [4.7,14.4] n = 11	7.7 [4.5,12.9] n = 13	5.4 [4.0,7.4] n = 37
Total	1.9 [1.0,3.5] n = 10	4.4 [3.0,6.6] n = 23	5.5 [3.7,8.3] n = 22	15.7 [12.9,19.0] n = 88	7.1 [6.1,8.3] n = 143

Any care (some or much)					

Women	2.6 [1.4,5.0] n = 9	8.4 [5.8,11.9] n = 27	7.9 [5.2,11.9] n = 21	29.6 [25.3,34.3] n = 116	13.1 [11.4,15.0] n = 173
Men	4.3 [2.2,8.3] n = 8	7.2 [4.3,11.8] n = 14	12.1 [7.6,18.9] n = 16	15.5 [10.8,21.8] n = 26	9.4 [7.4,11.8] n = 64
Total	3.2 [2.0,5.1] n = 17	7.9 [5.9,10.5] n = 41	9.3 [6.8,12.6] n = 37	25.4 [21.9,29.1] n = 142	11.8 [10.5,13.3] n = 237

### Correlates of dependency

Those with dependency needs were older, more likely to be female and widowed (Table 1). However, they were also less likely to be living alone and had a higher median household size. They had generally lower levels of education and (a non-significant trend) fewer household assets. Almost one in 10 of the whole sample, but only one dependent participant, continued to work. Dependent older people were also less likely to receive a government or occupational pension, but were no more likely than others to benefit from financial support from their family. Only six (2.5%) of those needing care were in receipt of a disability pension.

### Associations between impairments, diagnoses, disability and dependency

People with dementia accounted for 82 (57.3%) of those needing much care and 23 (24.5%) of those needing some care (Table 3). Other pathologies also clustered in the group needing much care – 72 (50.3%) had three or more limiting physical impairments, 49 (34.3%) had a current depressive episode and 42 (29.4%) reported having had a stroke. Evidently there was considerable comorbidity within this group, and comorbidity was strongly associated with needing higher levels of care. Dependency was very strongly associated with high levels of disability, and poor self-reported health.

**Table 3:** Health status, by level of dependency

	Needs much care	Needs some care	Does not need care	Whole sample	Statistical tests for trend (all 1 df)	p-value
*Health Conditions*						

Dementia	82 (57.3%)	23 (24.5%)	137 (7.7%)	242 (12.1%)	χ^2 ^= 316.3	<0.001
Three or more limiting physical impairments	72 (50.3%)	37 (39.4%)	355 (20.1%)	464 (23.1%)	χ^2 ^= 81.9	<0.001
Stroke	42 (29.4%)	14 (14.9%)	119 (6.7%)	175 (8.7%)	χ^2 ^= 88.4	<0.001
ICD 10 depressive episode	49 (34.3%)	15 (16.0%)	214 (12.1%)	278 (13.9%)	χ^2 ^= 51.4	<0.001

*Comorbidity*						

None of the above	15 (10.5%)	27 (28.7%)	1175 (66.4%)	1217 (60.6%)	χ^2 ^= 312.3	<0.001
1 only of the above	48 (33.6%)	49 (52.1%)	400 (22.6%)	497 (24.8%)		
2 of the above	50 (35.0%)	14 (14.9%)	160 (9.1%)	293 (14.6%)		
3 or more of the above	30 (21.0%)	4 (4.3%)	33 (1.9%)	67 (3.3%)		

*Disability*						

WHODAS II disability scale score (mean/SD)	58.2 (22.0)	33.0 (18.4)	12.3 (15.4)	16.5 (20.3)	F = 1168 Eta sq = 0.37	<0.001

*Subjective health*						

Bad or very bad	42 (29.4%)	12 (12.8%)	132 (7.5%)	186 (9.2%)	χ^2 ^= 93.4	<0.001

Poisson regression across the whole sample, adjusting for participant age, gender, education and marital status revealed that dementia (Prevalence Ratio 5.21, 95% confidence intervals 3.64–7.47), major depression (PR 1.88, 95% CI 1.31–2.69), stroke (PR 2.04, 95% CI 1.43–2.89) and physical illness (PR 2.18, 95% CI 1.58–3.01) were independently associated with needing much care. Population attributable prevalence fractions (the proportion of prevalent needs for care that might have been avoided had the condition been prevented) were for dementia 0.44, for physical illness 0.43 (stroke 0.16, three or more physical illnesses 0.27) and depression 0.16.

### The caregivers

For 214 of the 237 participants needing care (90.7%), the informant identified them self as the main caregiver; 190 (80.5%) were the main 'hands on' caregivers and 24 (10.2%) the main organisational caregivers. The largest group, 107 (45.2%), were children or children-in-law of the care recipient, 42 (17.7%) were spouses, 54 (22.8%) were other relatives, and 34 (14.4%) were friends or neighbours. One hundred and ninety (80.2%) of the caregivers were female, and 63 (26.5%) were in full or part-time employment. One hundred and three of the care recipients shared their household with one or more child under the age of 16 years (43.5%). None of these characteristics varied significantly between those with and without dementia.

### Dementia and dependency

Among those needing care, those with dementia had higher levels of disability and were more likely to need much care than others with dependency needs but no dementia (see Table 4). They were as likely to have three or more limiting physical illnesses and more likely to have reported a previous stroke. Their caregivers spent more time on general supervision and on assisting with basic activities of daily living – dressing, eating, bathing, toileting and grooming. Daytime and night time paid help was more likely to be required, but with borderline statistical significance. Caregivers of those with dementia experienced significantly more caregiver strain according to the Zarit Burden Interview.

**Table 4:** Characteristics of those needing care, care inputs and caregiver strain, by dementia status

	Those with dementia needing care N = 105	Others needing care N = 132	All those needing care N = 237	Statistical tests (all 1 df unless otherwise specified)	p-value
**Characteristics of those needing care**					

Three or more physical illnesses	44 (41.9%)	65 (49.2%)	109 (46.0%)	χ^2 ^= 1.3	0.26
Stroke	36 (34.3%)	20 (15.2%)	56 (23.6%)	χ^2 ^= 11.9	0.001
ICD 10 depressive episode	28 (26.7%)	36 (27.3%)	64 (27.0%)	χ^2 ^= 0.0	0.92
WHODAS II disability scale score (mean/SD)	57.3 (25.3)	41.0 (20.3)	48.2 (24.0)	T = -5.4 (194)	<0.001
NPI-Q Behavioural and psychological symptoms severity score (median/interquartile range) 1 MV	10 (5–17)	5 (2–9)	7 (3–12)	Z = -5.4	<0.001

**Care inputs**					

Needs much care	82 (78.1%)	61 (46.2%)	143 (60.3%)	χ^2 ^= 24.0	<0.001
Time per day spent supervising					
0	75 (71.4%)	123 (93.2%)	198 (83.5%)	χ^2 ^= 20.1	<0.001
1–5 hours	15 (14.3%)	9 (6.8%)	24 (10.1%)		
6 hours +	15 (14.3%)	0 (0%)	15 (6.3%)		
Time per day spent assisting with ADL (% spending more than one hour)					
Transport	4 (3.9%)	9 (6.8%)	13 (5.5%)	χ^2 ^= 0.03	0.86
Dressing	17 (18.2%)	11 (8.3%)	28 (11.8%)	χ^2 ^= 20.5	<0.001
Eating	21 (20.0%)	10 (7.5%)	31 (13.0%)	χ^2 ^= 13.1	<0.001
Looking after appearance	22 (20.9%)	13 (9.8%)	35 (14.7%)	χ^2 ^= 7.7	0.006
Toileting	23 (21.9%)	10 (8.6%)	33 (13.9%)	χ^2 ^= 21.4	<0.001
Bathing	27 (25.8%)	9 (6.8%)	36 (15.2%)	χ^2 ^= 30.2	<0.001

**Caregiver strain indicators**					

Caregiver psychological morbidity (SRQ>8)	32 (30.5%)	32 (24.2%)	64 (27.0%)	χ^2 ^= 1.2	0.28
Caregiver cut back on work to care	33 (31.4%)	31 (23.5%)	64 (27.0%)	χ^2 ^= 0.63	0.43
Caregiver Zarit Burden Interview score (mean/SD)	24.4 (16.6)	17.4 (13.8)	20.5 (15.0)	t = 3.6 (df = 232)	<0.001
Daytime paid help required	24 (22.9%)	17 (13.2%)	41 (17.5%)	χ^2 ^= 3.7	0.05
Night time paid help required	18 (17.1%)	10 (7.7%)	28 (12.0%)	χ^2 ^= 4.8	0.03
Additional informal support required	48 (46.2%)	49 (37.4%)	97 (41.3%)	χ^2 ^= 1.5	0.22

### Correlates of caregiver strain

We constructed a multivariate model examining the effects of dementia, depression and physical illness on caregiver strain, controlling for participant age, gender, education and marital status, and for the age, gender, marital status, relationship to participant and residence of the caregiver. Complete data was available for 195 of the 237 care recipient/caregiver dyads. Only dementia and age of participant (more strain in caregivers of younger participants) were associated with caregiver strain. In the fully adjusted model, dementia accounted for 6.9% of the variance in caregiver strain (F = 11.9, p = 0.001), depression 0.8% (F = 1.4, p = 0.25) and three or more physical impairments 0.5% (F = 0.9, p = 0.36). Including time spent assisting with activities of daily living in the model (as a potential mediating factor) marginally attenuated the strength of the association with dementia. Time spent assisting with activities of daily living accounted for just 2.2% of the variance in caregiver strain (F = 3.6, p = 0.06). Adding the severity of behavioural and psychological symptoms into the model abolished the association between dementia and caregiver strain. In the resulting model, behavioural and psychological symptoms alone accounted for 19.7% of the variance in caregiver strain (F = 38.7, p < 0.001).

## Discussion and conclusion

Surprisingly, this seems to be the first comprehensive, population-based study of dependency, and consequent care arrangements conducted in a low or middle income country. In Santo Domingo, 7.1% of the older population (aged 65 years or older) required much care and a further 4.7% required at least some care. The prevalence of dependency increased sharply with increasing age, with the more marked dependency needs being concentrated among those aged 80 years and over. Income insecurity was prominent among dependent older people, who were less likely to be in receipt of a pension and much less likely to work, but no more likely to receive financial support from their family. Needing much care was strongly associated with comorbidity between cognitive, psychological and physical health problems. However, dementia made the strongest independent contribution to needing much care, with a population attributable prevalence fraction of 0.44. Among those needing care, those with dementia stood out as being more disabled, as needing more care (particularly support with core activities of daily living), and as being more likely to need the additional support of a paid caregiver. Carers of those with dementia experienced more role strain than did carers of those with other underlying health conditions.

The strengths of our study are that we applied a one-phase survey methodology on a large representative sample of older adults, achieving a good response rate for a broad based assessment of health status (including cognitive, mental and physical disorders, global health and disability), social, demographic and economic circumstances. A suitable informant was interviewed for every participant to ascertain needs for care directly (as opposed to merely inferring this from participant reports of limitations in activities of daily living). In the event of the participant being identified as a care recipient, we obtained further detailed information on the care arrangements, and any consequent strain experienced by the caregiver. The main weakness is that dependency was ascertained using a semi-structured interview. The rating of level of dependency (some care versus much care) was somewhat subjective. This was intentional, given the difficulties of developing a more structured approach that would have had equal validity across many different countries and cultures. Nevertheless, data on inter-rater reliability would have been valuable. Also, dementia and depression were diagnosed on the basis of extensive structured clinical interviews whereas stroke and other physical impairments were assessed only on the basis of self-report. Therefore, there may have been greater misclassification of the physical health outcomes, possibly leading to an underestimation of their contribution to dependency. Conversely we did not specify, when screening for dependency, that care could include prompting, remembering and supervising, in addition to physical care. This might have led to an underestimation of the contribution of dementia to dependency.

Our estimate, of 11.8% of participants with dependency needs is a little lower than that from population-based surveys of those aged 65 and over from England and Wales [26] (15.7% with significant disability among whom 86% had dependency needs), Scotland [27] (15% with short interval dependency), Spain [28] (15.5% with dependency in one or more of seven ADLs), France [29] (12.4% confined to home or bed) and the USA National Long Term Care surveys [30] (17.1% disabled in one or more activities of daily living, or living in a care home). Proper comparison would require age adjustment. Only the last of these studies provides age-specific prevalence estimates (65–74 8.4%; 75–84 21.4%; 85 and over 52.7%). Applying these prevalences to the age structure of our sample gives 377.5 expected cases of dependency versus the 237 that we observed, a standardised morbidity ratio of 62.8.

People with dementia (often comorbid with other health conditions) accounted for more than half of those needing much care, and a quarter of those needing some care. Our findings regarding the particular character of the experiences linked to caring for an older person with dementia are consistent with the only comparable findings from a population based survey, that of more than 1,500 family caregivers interviewed in the 1996 USA National Caregiver Survey [31]. In the USA, dementia caregivers spent longer providing care, reported more impacts on their employment, more caregiver strain, more mental and physical health problems, less time for leisure and more family conflict. Unfortunately, dementia, which has a uniquely devastating impact on capacity for independent living is often forgotten when policies for prevention and management of NCDs are proposed, as for example with the recent Lancet Series on non-communicable diseases [32], and the WHO's Global Report on Innovative Care for Chronic Conditions [33]. An original finding from our analysis is that the effect of dementia upon caregiver strain is entirely mediated by the severity of behavioural and psychological symptoms. Furthermore the independent predictive power of these symptoms (19.6% of the variance in caregiver strain explained) was much greater than that of dementia (6.9% of the variance). Although often referred to as behavioural and psychological symptoms of dementia (BPSD) these features (psychological symptoms – delusions, hallucinations, depression/dysphoria, anxiety, elation/euphoria; behavioural symptoms – apathy, disinhibition, agitation/aggression, motor, sleep and appetite disturbance) are not condition specific [24,34].

LAMIC governments will face many challenges in the near future as numbers of dependent older people increase rapidly. An adequate response will require

a) policies to prevent disability through the control of NCDs,

b) policies to limit disability through more active community-based rehabilitation,

c) policies to manage disability and dependency through improved access to age-appropriate long-term support and care.

Such measures are already strongly advocated through international agreements including the Madrid International Plan on Action on Ageing (2002) and the UN Convention on the Rights of People with Disabilities (2007). A recent WHO report on long-term care policy [35] noted wide international variation in the apportioning of responsibilities between families and the state, but proposed that all countries could and should determine transparently the assistance needed by older people and their carers, and the eligibility for and financing of long-term care support. In practice, LAMIC governments have avoided providing or financing long-term care [36], and few if any have comprehensive policies and plans.

Primary health care services in LAMIC tend to fail older people with dementia, [36-39] as they focus on acute 'treatable' conditions and are clinic-based. There is a need for a paradigm shift beyond simple curative interventions to encompass long-term regular support, community outreach and domiciliary care, family involvement and multisectoral working. The WHO have proposed an alternative care framework (Innovative Care for Chronic Conditions)[33] that addresses many of these issues. ICCC is likely to be as applicable to dementia care as to other chronic NCDs – mental disorders, stroke, heart disease and diabetes – for which it was initially proposed. 10/66 is currently testing the effectiveness of training community healthcare workers to identify people with dementia [40-42], and to deliver a brief intervention to educate and train caregivers [10]. In practice, such interventions will need to be incorporated into horizontally constructed programs addressing the generic needs of frail, dependent older people and their caregivers, whether arising from cognitive, mental or physical disorders. One focus should perhaps be upon the assessment and syndromal management of the behavioural and psychological problems that, our research suggests, contribute so much to caregiver strain.

A key finding from our analysis is the economic disadvantage experienced by older dependent persons and the families that care for them. For older people in developing countries 'dependency anxiety' [37,43,44] – not wanting to be a burden on relatives, fearing inadequate support, and therefore wishing to maintain independence from the family – is a key motivating principle. The Nobel Prize winning economist Joseph Stiglitz commented:

'There is no subject of greater importance than the ageing of the population and the provision of social protection for older people. It affects the very nature of our societies and concerns not only older people, but all sections of the population.' [45].

Some LAMIC governments have sought to encourage or coerce families to shoulder this responsibility [36]. For example, the Indian parliament passed a law to this effect in 2007. Such policies seem destined to fail in the longer-term. Inexorable trends towards more internal and international migration, declining fertility, higher levels of education and increased participation of women in the workforce will reduce the availability and willingness of children (principally daughters and daughters-in-law) to care [36]. Social pensions provide insurance against the risks that older people face, including uncertainty over how long they will live, how long they will remain healthy, whether they can count upon the support of others if they need it, and how long they can earn an income. Furthermore, in rural Brazil, they have been shown to support whole families [46], reducing the risk of household poverty [47], and have even been linked to increased school enrolment, particularly of teenage girls [45]. Most importantly they reinforce reciprocal family ties, changing the perspective from one in which older people are seen as a dependent drain upon household resources to one in which they can be properly valued for their non-economic as well as their economic contributions. Dependent older people would be particularly likely to benefit – informal care would be bolstered and formal/paid care would be more affordable.

## Competing interests

The 10/66 Dementia Research Group works closely with Alzheimer's Disease International (ADI), the non-profit federation of 77 Alzheimer associations around the world. ADI is committed to strengthening Alzheimer associations worldwide, raising awareness regarding dementia and Alzheimer's Disease and advocating for more and better services for people with dementia and their caregivers. ADI is supported in part by grants from GlaxoSmithKline, Novartis, Lundbeck, Pfizer and Eisai. DA is the Chair-elect of ADI.

## Authors' contributions

MP conceived and performed the analysis and drafted the manuscript. RR assisted in the analysis and helped to draft the manuscript. DA, GR and CP participated in the design and coordination of the study and helped to draft the manuscript. All authors read and approved the final manuscript.

## Pre-publication history

The pre-publication history for this paper can be accessed here:


